# Organic–Inorganic Eu^3+^/Tb^3+^ codoped hybrid films for temperature mapping in integrated circuits

**DOI:** 10.3389/fchem.2013.00009

**Published:** 2013-07-08

**Authors:** Carlos D. S. Brites, Patrícia P. Lima, Nuno J. O. Silva, Angel Millán, Vitor S. Amaral, Fernando Palacio, Luís D. Carlos

**Affiliations:** ^1^Departamento de Física and CICECO, Universidade de AveiroAveiro, Portugal; ^2^Departamento de Fisica de la Materia Condensada, Facultad de Ciencias and Instituto de Ciencia de Materiales de Aragón, CSIC–Universidad de ZaragozaZaragoza, Spain

**Keywords:** organic–inorganic hybrids, lanthanide ions, molecular thermometer, spatio-temporal resolution

## Abstract

The continuous decrease on the geometric size of electronic devices and integrated circuits generates higher local power densities and localized heating problems that cannot be characterized by conventional thermographic techniques. Here, a self-referencing intensity-based molecular thermometer involving a di-ureasil organic-inorganic hybrid thin film co-doped with Eu^3+^ and Tb^3+^ tris (β-diketonate) chelates is used to obtain the temperature map of a FR4 printed wiring board with spatio-temporal resolutions of 0.42 μm/4.8 ms.

## Introduction

Miniaturization, integration and the increase of physical complexity in electronic devices and circuits tend to generate higher local power densities and localized heating problems (Burzo et al., 2005; Christofferson et al., 2008). The management of the heat flux generated by the several billion of transistors that actually exist in a single chip is one of the main challenges of the modern electronics industry. Thermal management is therefore essential to improves electronics performance and reliability, posing a limitation stronger than the downscaling itself. The temperature distribution across integrated circuits must be, then, accurately mapped with superior spatial resolution (Burzo et al., 2005; Yarimaga et al., 2011; Liu et al., 2012).

The well-known limitations of contact thermometers has strengthened the development of non-contact thermometry techniques (Brites et al., 2012), such as, infrared (IR) thermography (Meola and Carlomagno, 2004), thermoreflectance (Kolodner and Tyson, 1983; Christofferson et al., 2008), optical interferometry (Kersey and Berkoff, 1992), Raman spectroscopy (Frazão et al., 2009) and luminescence (Aigouy et al., 2005; Brites et al., 2010; Vetrone et al., 2010; Kuzmin et al., 2011; Yarimaga et al., 2011; Benayas et al., 2012). Luminescence methods combine temperature sensitivities up to 5.0%·K^−1^ with spatial resolution below 1 μm and have been used to monitor and map temperature on integrated circuits and electronic devices (Aigouy et al., 2005; Jung et al., 2011; Yarimaga et al., 2011; Brites et al., 2013).

Here we report the use of a self-referencing intensity-based molecular thermometer involving a di-ureasil organic-inorganic hybrid thin film co-doped with Eu^3+^ and Tb^3+^ tris (β-diketonate) chelates to map the temperature over a FR4 printed wiring board using commercial detectors and excitation sources.

## Materials and methods

**Figure 1:**
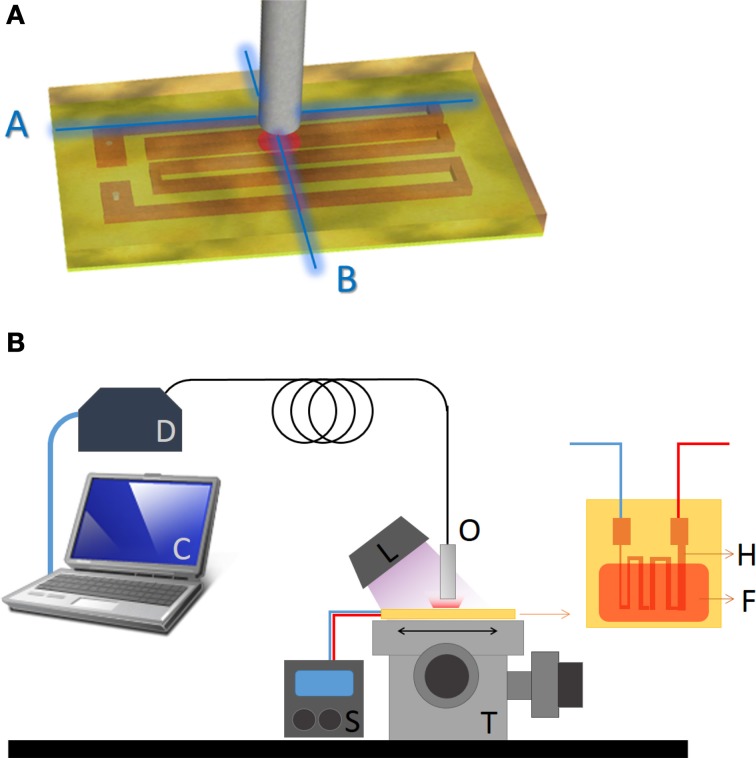
**(A)** Geometry of the FR4 printed wiring board where A and B characterize the directions used in temperature mappings. **(B)** Schematic representation of the setup used. The heating circuit (H) was covered with a UET–1.3 film (F) and positioned over a translation stage (T). The temperature was controlled by the source (S). A handheld UV lamp (L) was used to excite the film. The optical fiber (O) was connected to the portable detector (D) and the emission spectra were processed in the computer (C).

The synthesis and characterization of the Eu^3+^/Tb^3+^ co-doped di-ureasil organic/inorganic hybrids has already been described in detail elsewhere (Brites et al., 2010, 2013). The first step of the synthesis involves the formation in tetrahydrofuran of an urea cross-linked hybrid precursor (De Zea Bermudez et al., 1999). In the second step, the [Eu(btfa)_3_(MeOH)(bpeta)] and [Tb(btfa)_3_(MeOH)(bpeta)] complexes [where btfa^−^ is 4,4,4-trifluoro-l-phenyl–1,3-butanedionate, bpeta is 1,2–bis(4–pyridyl)ethane and MeOH methanol] were incorporated as ethanolic solutions together with water and HCl to promote the hydrolysis of the urea cross-linked hybrid precursor. The di-ureasil thermometer, hereafter named UET–1.3, incorporates the Eu^3+^ and Tb^3+^ β-diketonate complexes in a 1:3 mass proportion, respectively, and is processed as a film or a monolith. Although the films can be obtained with high thickness control by dip- or spin-coating techniques, here a ~10 μm thermosensitive UET–1.3 layer was deposited over a FR4 printed circuit board (Figure 1A). UET–1.3 monoliths were also employed for photoluminescence characterization and temperature calibration.

The size (250–1000 μm) and geometry of the copper tracks etched over the FR4 printed wiring board determine the temperature distribution profile that is controlled by the current feeding the board. The UET–1.3 film was excited with a handheld UV lamp (Spectroline E-Series, Aldrich, Model Z169625, operating at 365 ± 25 nm) and the emission was collected with optical fibers of 200 and 450 μm inner diameter. The emission spectra were then analyzed in a portable spectrometer (Ocean Optics, USB-4000 FL) with limit of detection of 1/200. The fiber was positioned over the circuit that was moved with a nanomax 3-Axis flexure translation stage from ThorLabs®, with variable steps (ranging from 800 to 50 μm).

A key parameter to evaluate the performance of a luminescent thermometer is the relative sensitivity (*S*), defined as the relative variation on the thermometric parameter (*Q*) with temperature:

(1) $$S=\frac{dQ/dT}{Q}.$$

The relative sensitivity was proposed as a figure of merit to compare the performance of distinct thermometers (Brites et al., 2012). When the temperature is accessed in different points it is possible to define the spatial resolution of the measurement (δ*x*) as the minimum distance between points that present temperature higher than the temperature uncertainly (δ*T*). It can be estimated using the common expression (Kim et al., 2012):

(2) $$\delta x=\frac{\delta T}{{\left|\vec{\nabla }T\right|}_{\max}}$$

where ${\left|\vec{\nabla }T\right|}_{\max}=dT/dx$ is the maximum temperature gradient of the mapping. The experimental setup used defines the temperature detection limit and the temperature gradient. The temporal resolution of the measurement (δ*t*) is the minimum time interval between measurements presenting temperature higher than δ*T*:

(3) $$\delta t=\frac{\delta T}{dT/dt}$$

where *dT/dt* is the temperature change per unit of time.

## Results

**Figure 2:**
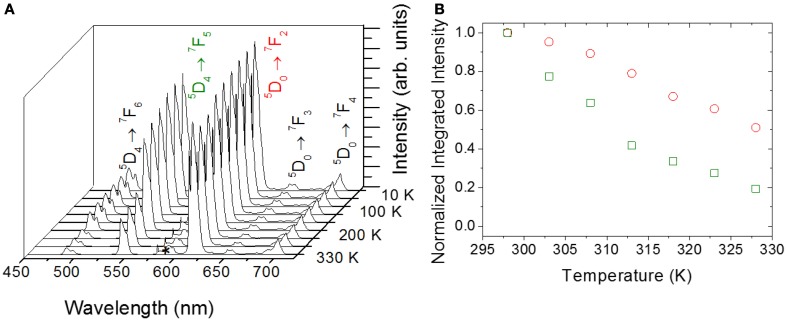
**(A)** Temperature dependence of the emission spectra of UET–1.3 (excited at 365 nm) in the 10–330 K temperature range. The f-f lines corresponding to the ^5^D_4_→^7^F_6, 5_ (Tb^3+^) and ^5^D_0_→^7^F_2-4_ (Eu^3+^) transitions are identified. In the area marked with an asterisk there is a superposition between the emission of Eu^3+^ (^5^D_0_→^7^F_0, 1_) and Tb^3+^ (^5^D_4_→^7^F_4_). **(B)** Normalized integrated intensity of ^5^D_0_→^7^F_2_(red) and ^5^D_4_→^7^F_5_(green) in the temperature range 290–330 K.

The emission spectra (Figure 2A), as well as the ^5^D_4_ and ^5^D_0_ lifetime values, are temperature dependent in the 10–330 K range (Brites et al., 2010). This dependence and the room-temperature emission quantum yield value (0.16 ± 0.02 at 365 nm) permit to anticipate that the UET–1.3 thermometer sensitivity in the 290–330 K temperature range is enough to implement a sensor based on the analysis of the emission spectra using commercially cost-effective excitation sources and detectors (e.g., hand-held UV lamp and a portable optical-fiber connected detector, Figure 1B).

**Figure 3:**
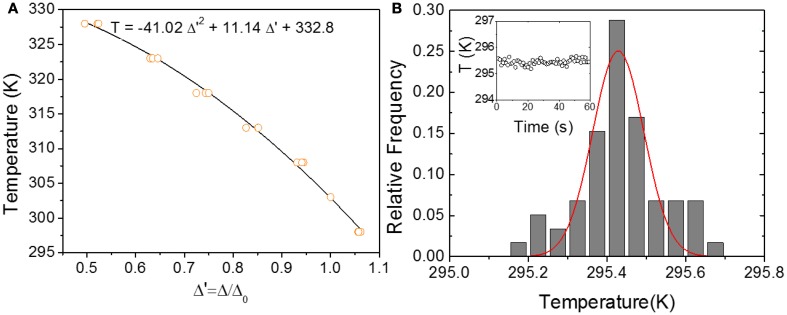
**(A)** Local calibration curve of UET–1.3 for the temperature range 290–330 K. The temperature was cycled three times and the emission spectra recorded at equal time intervals when the temperature increases. For all emission spectra the computed Δ parameter was divided by its value at 303 K (Δ_0_) to get a thermometric parameter near the unit. The results for all cycles are overlapped and the second degree polynomial fit presented in the Figure (*r*^2^ > 0.995) is the local calibration curve. **(B)** Relative frequency of the UET–1.3 temperature read during 60 s in the absence of current in the heating circuit (inset). The distribution of temperature (vertical bars) was fitted to a Gaussian (solid line) resulting in a maximum at 295.4 K and a full- width-at-half-maximum of 0.15 K.

The temperature of the UET–1.3 film can be accessed measuring the emission spectra and using the thermometric parameter Δ, defined as:

(4) $$\Delta =I_{Eu}^{2}-I_{Tb}^{2},$$

where *I*_*Eu*_ and *I*_*Tb*_ stands for the integrated areas of the ^5^D_0_→^7^F_2_ and ^5^D_4_→^7^F_5_ transitions, assigned to Eu^3+^ and Tb^3+^, respectively (Figure 2B). Other definitions for the thermometric parameter are also plausible (namely the *I*_*Tb*_/*I*_*Eu*_ ratiometric form) without losing the generality of the method. Here we use, however, the previously reported thermometric parameter Δ (Brites et al., 2010, 2011, 2013). The UET–1.3 local calibration curve (Figure 3A) for the temperature range 290–330 K was computed by three consecutive temperature cycles. A second order polynomial fit to the experimental Δ values allows the conversion of intensities into temperature. Given the 1/200 detection limit of the detector used in the experiments we can anticipate an ultimate temperature detection limit of δ*T*_*min*_ = 0.01 K. Nevertheless, in the experimental conditions used, a temperature uncertainly δ*T* = 0.15 K was estimated from the full-width-at-half-maximum value of the temperature reads Gaussian distribution in the absence of any external heat source (Figure 3B).

**Figure 4:**
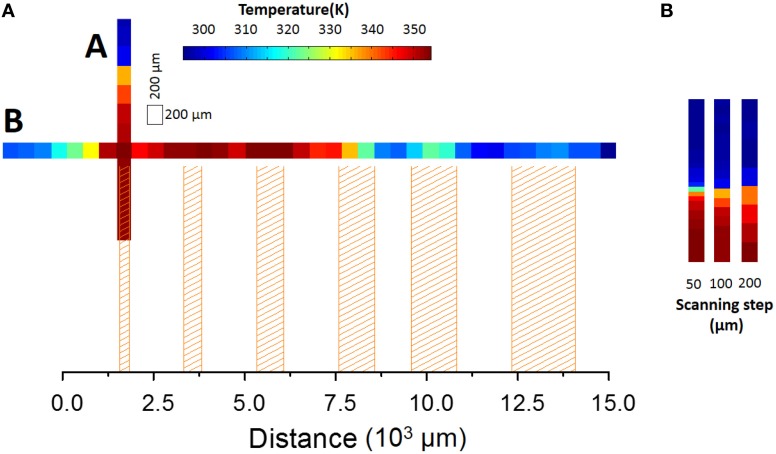
**Pseudocolor temperature maps reconstructed from the emission of UET–1.3 collected with the 200 μm core diameter fiber (A) along the directions A and B indicated in Figure 1A, using a scanning step of 200 μm, and (B) along A direction, changing the scanning step from 50 to 200 μm.** The shadowed areas correspond to the position of the copper tracks.

**Figure 5:**
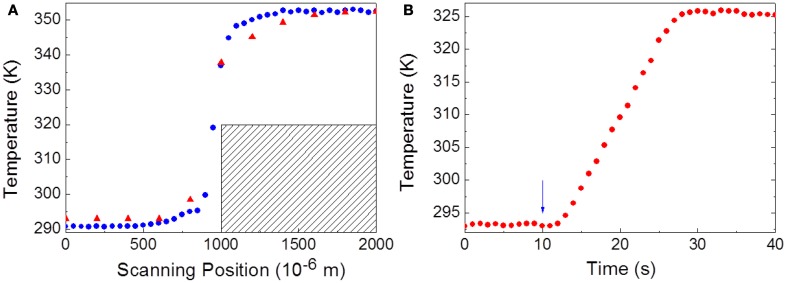
**(A)** Temperature profiles along the A direction indicated in Figure 1A for the 200 μm core diameter fiber using scanning steps of 50 μm (blue squares) and 200 μm (red circles). The maximum temperature gradient is 0.357 × 10^6^ K·m^-1^. **(B)** Temporal dependence of the temperature over the copper track represented in **(A)** measured with the 450 μm core diameter fiber. The arrow marks the instant when the heating current was turned on.

**Figure 6:**
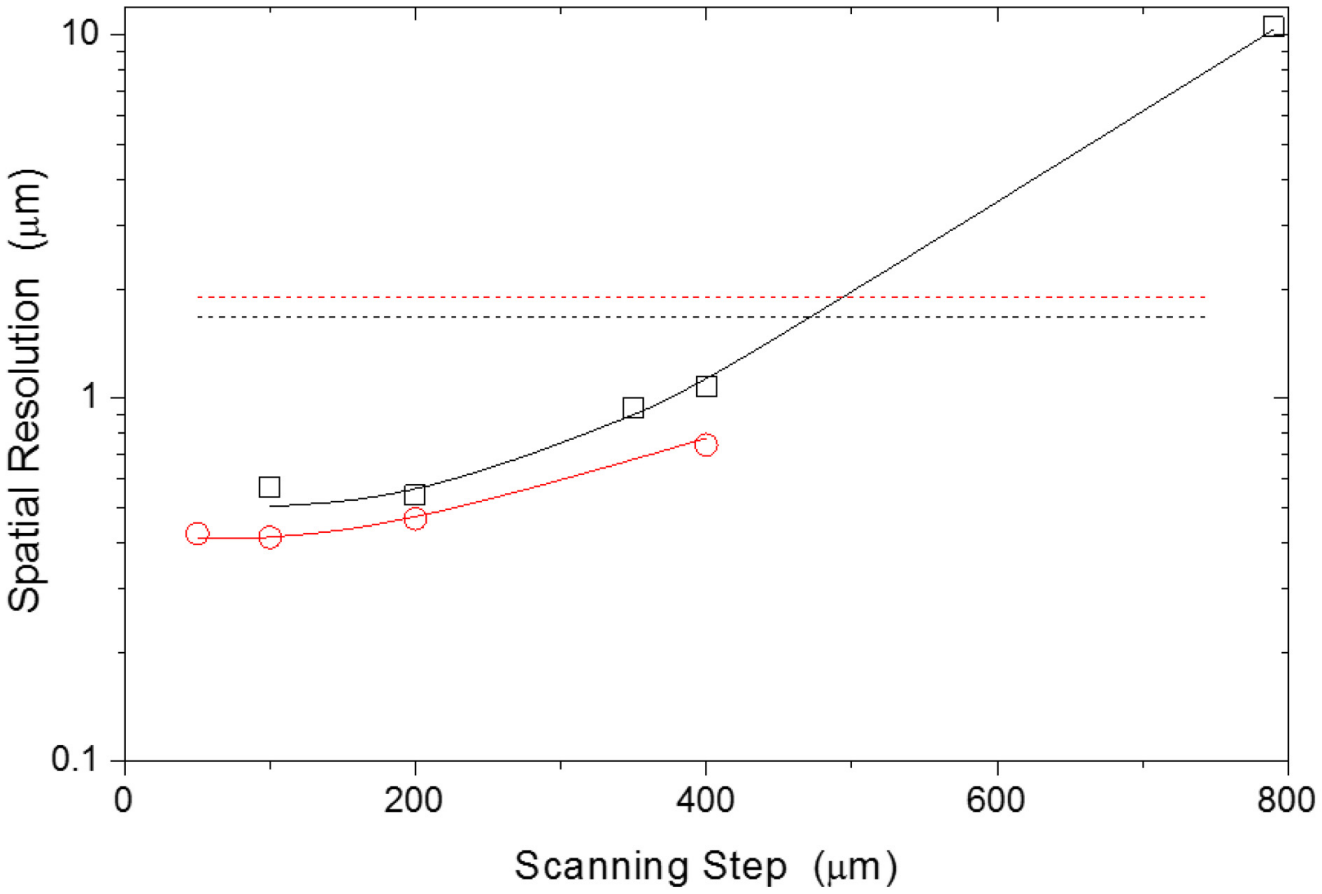
**Spatial resolution of the UET–1.3 thermometers for different scanning steps.** The red circles and black squares correspond to the mappings using the 200 and 450 μm diameter fiber, respectively. The solid lines are guides for the eyes. The interrupted red and black lines correspond to the Rayleigh spatial resolution limit of the 200 and 450 μm diameter fibers, respectively.

Using a fixed heating current, bi-dimensional temperature profiles of the FR4 printed wiring board along the directions A and B in Figure 1A were reconstructed from the emission spectra of UET-1.3 using the 200 and the 450 μm core diameter fibers and different scanning steps (ranging from 800 to 50 μm), Figure 4. The spatial resolution of the thermometer was calculated by Equation 2 using the temperature profiles along the A direction in Figure 1A (Figure 5A). The results are compared in Figure 6. The calculated spatial resolution is considerably improved from 10 to 0.5 μm when the scanning step is decreased from 800 μm to values around the fiber inner radius (200 and 100 μm, for the 450 and 200 μm fibers, respectively). For lower scanning step values the spatial resolution remains almost constant. The higher spatial resolution measured with the 200 μm core diameter fiber is 0.42 μm, a value 4.5 times lower than the Rayleigh limit of diffraction (1.89 μm) in the experimental conditions used (see discussion below). Despite the minor changes of the spatial resolution when the scanning step decreases from 200 to 50 μm (Figure 6), the transition from the low to high temperature regions in the profile along the A direction in Figure 1A is clearly much more defined for 50 μm (Figure 4B).

For the determination of the temporal resolution limit of the mapping, δ*t*, the heating current was turned on and the thermalisation of the copper track followed using the UET–1.3 thermometer readout (Figure 5B). The temporal resolution achieved, 4.8 ms, is of the same order of magnitude than the detector integration time set (e.g., 10 ms).

## Discussion

The seminal work of Kolodner and Tyson (1983) demonstrated the potential of non-contact thermometry to map integrated circuits reporting the temperature mapping of a MOSFET through the emission of a polymer film containing Eu(tta)_3_2H_2_O (tta^−^ stands for thenoyltrifluoroacetonate). A 0.01 K temperature resolution is expected based on shot noise of the collected light; however experimental conditions (e.g., electric fluctuations of detection system) degrade this limit to the 0.1–1.0 K range. The spatial resolution is limited by the CCD smoothing to 15 μm (Kolodner and Tyson, 1983). Since 2005 there is a significant number of references reporting the temperature mapping of active electronic devices with high spatial resolution.

Burzo et al. (2005) used the thermoreflectance of a MOSFET device to map the temperature in a window of 15 × 50 μ m, with uncertainly of 13% (or 2.6 K) and spatial/temporal resolutions of 2.3 μm/1.1 μ s, respectively. Tessier et al. (2007) recognized that the thermoreflectance of integrated circuits fabricated over silicon can also be used in backside imaging exploiting the transparency of this substrate in the near IR region. The technique produces temperature mapping with spatial resolution of 1.7 μm and temporal resolution defined by the camera triggering at 50 ms (Tessier et al., 2007). The small value and the temperature and wavelength dependence of the thermoreflectance coefficient is actually the most challenging aspect for thermoreflectance based thermometry, making the setup for temperature mapping quite sophisticated (Burzo et al., 2005).

The use of a scanning thermal microscope (SThM) adapted for fluorescence reads (Benayas et al., 2012) was reported by Aigouy et al. (2005, 2009, 2011) and Saïdi et al. (2009, 2011) to work in the sub-wavelength spatial resolution regime. Regarding high-resolution thermal imaging of integrated circuits, Saïdi et al. (2011) used a small fluorescent Er^3+^/Yb^3+^-doped PbF_2_ nanocrystal as a temperature sensor. The technique presents temperature uncertainly ~1.0 K, spatial resolution of 0.027 μm, despite the relatively long acquisition times (100 ms per pixel), that invalidates the transient mapping of the device (Saïdi et al., 2011).

In fact, the use of conventional optical microscopy for temperature mapping set the Rayleigh criterion as the ultimate spatial resolution limit (Tessier et al., 2007; Serrels et al., 2008):

(5) $$\delta x_{RL}=1.22\frac{y\lambda }{D}=1.22\frac{\lambda }{NA}$$

where λ is the maximum wavelength, *y* is the distance to the emitting surface, *D* is the diameter of the detector and *NA* is the correspondent numerical aperture. In recent years, a number of fluorescence imaging techniques with sub-diffraction-limit resolution have been developed, achieving a spatial resolution until 0.01 μm (Rust et al., 2006).

The Rayleigh spatial resolutions applied to the experimental parameters used in this work are presented in Figure 6. Contrary to the Rayleigh spatial limit that increases for narrower fibers, the temperature spatial resolution as defined here (Equation 2) improves when narrower fibers are used. This conclusion results from the area probed by the fiber (fiber field-of-view) can change significantly in a small step (the area change is 1.25% of the fiber field-of-view for the best spatial resolution value). As the temperature readout results of a spectroscopic measurement (the temperature at each position is averaged over the field-of-view of the fiber) it is not limited by the Rayleigh criterion. The spatial resolution is limited by the experimental setup used that produces a field-of-view averaged temperature change above the sensitivity of the detector.

**Figure 7:**
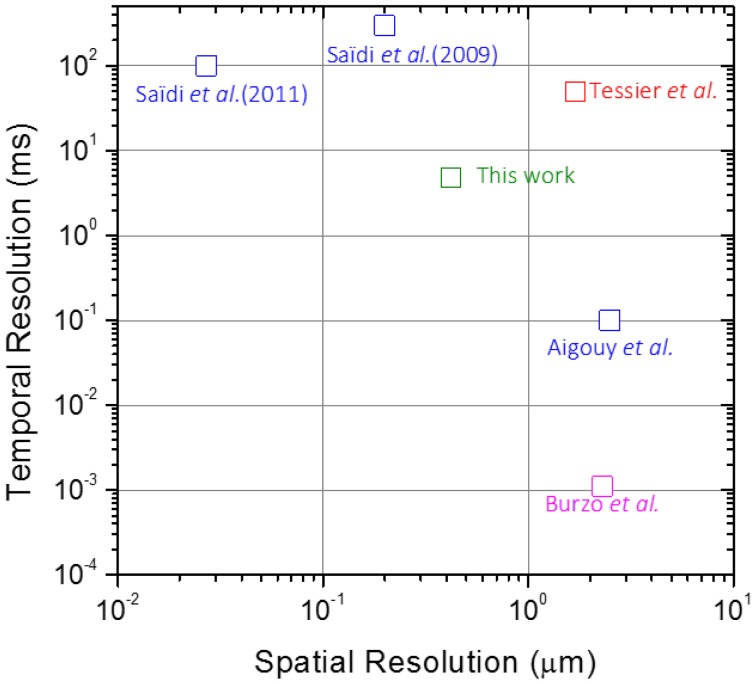
**Spatio-temporal resolution for temperature mapping of integrated circuits using distinct luminescent thermometers**.

Figure 7 compares the spatio-temporal resolution for temperature mapping of integrated circuits using distinct luminescent thermometers. This figure shows that the choice of high spatial resolution compromises the temporal resolution and vice-versa. To reach spatial resolution in the micrometer range (1–10 μm) the temporal resolution ranges from the microsecond to the fraction of the second. Although thermoreflectance based technique displays high temporal resolution and SThM based techniques present the highest spatial resolution, the combination of high spatial and temporal resolutions in a single temperature mapping has not been reported yet, showing that there is plenty of room to improve the temperature mappings reported so far.

## Conclusions

Here we report the use of Eu^3+^/Tb^3+^ co-doped di-ureasil thin films to perform temperature mapping of integrated circuits with temperature with spatio-temporal resolution of 0.42 μm/4.8 ms. The finer temperature spatial resolution reached is below the Rayleigh optical spatial resolution limit, because it results from a spectroscopic measurement. The temperature mapping spatial resolution depends on the scanning step and on the diameter of the optical fiber used. The spatial resolution is not improved for scanning step values lower than the fiber inner radius; nevertheless narrower fibers produce finer temperature spatial resolution.

The technique presented here can be easily used for routine temperature maps with cost-effective equipment (e.g., a portable spectrometer and a handheld excitation source) furnishing results of the same order of magnitude of those obtained with more sophisticated setups. We can foresee that the spatio-temporal resolution values presented here does not constitute the ultimate limit of the technique that predictably will improve both resolutions using sensitivity-enhanced materials, for the 290–330 K operating range. Although the UET–1.3 film thermometer is a cost effective competitive approach combining equitable spatial and temporal resolution we are currently investigating new materials to address these demands.

### Conflict of interest statement

The authors declare that the research was conducted in the absence of any commercial or financial relationships that could be construed as a potential conflict of interest.
